# Optimization of *Streptococcus agalactiae* Biofilm Culture in a Continuous Flow System for Photoinactivation Studies

**DOI:** 10.3390/pathogens10091212

**Published:** 2021-09-18

**Authors:** Michal K. Pieranski, Michal Rychlowski, Mariusz Grinholc

**Affiliations:** 1 Laboratory of Photobiology and Molecular Diagnostics, Intercollegiate Faculty of Biotechnology University of Gdansk and Medical University of Gdansk, 80-307 Gdansk, Poland; mariusz.grinholc@biotech.ug.edu.pl; 2 Laboratory of Virus Molecular Biology, Intercollegiate Faculty of Biotechnology University of Gdansk and Medical University of Gdansk, 80-307 Gdansk, Poland; michal.rychlowski@biotech.ug.edu.pl

**Keywords:** *S. agalactiae*, Group B *Streptococcus* (GBS), biofilm, continuous flow, Center for Disease Control and Prevention (CDC) Biofilm Reactor, photoinactivation, Rose Bengal

## Abstract

*Streptococcus agalactiae* is a relevant cause of neonatal mortality. It can be transferred to infants via the vaginal tract and cause meningitis, pneumonia, arthritis, or sepsis, among other diseases. The cause of therapy ineffectiveness and infection recurrence is the growth of bacteria as biofilms. To date, several research teams have attempted to find a suitable medium for the cultivation of *S. agalactiae* biofilms. Among others, simulated vaginal fluid has been used; however, biofilm production in this medium has been found to be lower than that in tryptic soy broth. We have previously shown that *S. agalactiae* can be successfully eradicated by photoinactivation in planktonic culture, but there have been no studies on biofilms. The aim of this study was to optimize *S. agalactiae* biofilm culture conditions to be used in photoinactivation studies. We compared biofilm production by four strains representing the most common serotypes in four different broth media with crystal violet staining. Then, we evaluated stationary biofilm culture in microtiter plates and biofilm growth in a CDC Biofilm Reactor^®^ (BioSurface Technologies, Bozeman, MT, USA) under continuous flow conditions. Subsequently, we applied Rose Bengal-mediated photoinactivation to both biofilm models. We have shown that photoinactivation is efficient in biofilm eradication and is not cyto/phototoxic to human keratinocytes. We found conditions allowing for stable and repetitive *S. agalactiae* biofilm growth in continuous flow conditions, which can be successfully utilized in photoinactivation assays and potentially in all other antibacterial studies.

## 1. Introduction

*Streptococcus agalactiae*, the most common representative of Group B *Streptococcus* (GBS), is a relevant cause of neonatal mortality. Approximately 10–30% of women carry *S. agalactiae* in their vaginal tract, from which it can be transferred to infants during labor and cause meningitis, pneumonia, arthritis, or sepsis, among other diseases. Prenatal antibiotic prophylaxis has significantly decreased the incidence of newborn infections in the Western Hemisphere, but there are still parts of the world where this procedure is not routine [1]. Worrying issues are the increasing incidence of antibiotic resistance among GBS, such as to penicillin G or macrolides, which are first-line therapies. In our study, we chose the most frequently represented capsular serotypes in Poland, which are IA, III, and V [2]. These serotypes are also the most common cause of urinary tract infections among adults [3]. Apart from antibiotic resistance, a significant cause of persistence and recurrence of infections such as bacterial vaginosis is biofilm production [4]. Additionally, bacteria grown as biofilms are significantly less susceptible to antibiotics than bacteria grown in planktonic culture. The minimum biofilm eradication concentration (MBEC) of penicillin G against *S. agalactiae* is approximately 500 times higher than the minimum inhibitory concentration (MIC) effective against planktonic culture [5]. As the physiological vaginal pH is below 4.5 and *S. agalactiae* serotypes III and V form strong biofilms under acidic conditions, it is important to evaluate the effectiveness of the proposed therapies in a biofilm model [6].

To date, several research groups have attempted to find a suitable medium for the cultivation of *Streptococcus* biofilms. Among others, simulated vaginal fluid has been used. However, in the case of *S. agalactiae*, biofilm production in simulated vaginal fluid has been found to be lower than that in tryptic soy broth (TSB) [7]. Biofilm formation has also been found to be greater in TSB than in Todd Hewitt broth (THB), Luria Bertani broth (LB), or brain–heart infusion broth (BHI) [8]. For these reasons, we decided to evaluate *S. agalactiae* biofilm formation in other broths used for the cultivation of vaginal physiological flora or vaginal pathogens. New York City III (NYC) broth has been used in the culture of *Trichomonas vaginalis* and biofilms of *Gardnerella sp., Atopobium vaginae, Lactobacillus iners, Mobiluncus curtisii, Peptostreptococcus anaerobius*, and *Prevotella bivia* [9,10]. Schaedler broth has been used in biofilm cultures of *Gardnerella vaginalis* [11]. De Mann, Rogosa, and Sharpe broth (MRS) has been used in biofilm cultures of *Lactobacillus plantarum*, *Lactobacillus crispatus*, *G. vaginalis*, and *P. bivia* [12,13,14].

With increasing antibiotic resistance, there is a tremendous demand for alternative antibacterial therapy development. In our research, we propose antimicrobial photodynamic inactivation (aPDI). aPDI requires the simultaneous presence of photosensitizing molecules, light, and oxygen. During the aPDI process, reactive oxygen species (ROS) are created inside or in close proximity to the bacterial cell. This phenomenon leads to damage to proteins, nucleic acids, and lipids and ultimately to bacterial death [15]. Rose Bengal (RB) is a photosensitizing dye efficient in inactivation of both planktonic and biofilm cultures of, i.e., *Staphylococcus aureus*, *Listeria innocua*, *Enterococcus hirae and Escherichia coli* [16,17]. It is characterized by the most efficient single oxygen generation of xanthene dyes and high water solubility [18]. Moreover, it is considered a molecule with a low level of cytotoxicity and high biocompatibility [19]. We have already shown that *S. agalactiae* can be successfully eradicated with RB-mediated aPDI in planktonic culture, but there have been no studies about the photoinactivation of *S. agalactiae* biofilms [20]. Therefore, the aim of this study was to optimize *S. agalactiae* biofilm culture conditions so that an assay system can be used in photoinactivation studies and potentially in all other antibacterial treatment tests.

## 2. Results

### 2.1. Microtiter Plate Biofilm Culture and Crystal Violet Staining Revealed Differences in Biofilm Production among the Studied Strains

The first stage of biofilm culture optimization was to screen for strong biofilm producers among the studied *S. agalactiae* strains using the most commonly applied microtiter plate method and crystal violet (CV) staining. A comparison of biofilm growth in four chosen broths was performed. For each broth, the biofilm was cultured in 96-well plates with full-strength or 2-fold diluted broth. After 24 h of growth, CV staining was performed. In all investigated broths, serotype V (s. V) strain produced a weak biofilm. Strains expressing serotypes IA and III (s. IA, s. III) as well as strain ATCC 27956 were characterized as moderate and strong biofilm producers according to particular broths. For both TSB and MRS broths, biofilm growth was stronger in diluted broth than in full-strength broth. A similar effect was also observed for the s. IA strain in NYC broth, and in Schaedler broth, growth intensity did not depend on broth concentration. For the s. III and ATCC 27956 strains, biofilm growth was strong at both concentrations of the NYC and Schaedler broths (Table 1). Therefore, these two strains, i.e., s. III and ATCC 27956, were used for further investigation.

**Figure 1:**
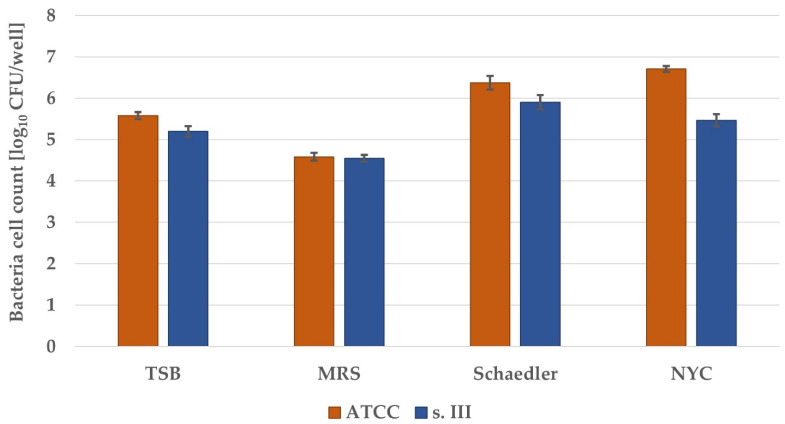
Growth of *S. agalactiae* biofilm cultures on microtiter plates. Overnight cultures were diluted in appropriate medium and incubated for 4 h. Then, the broth was changed, and incubation continued for 20 h. Dispersed biofilms were plated, and colonies were enumerated. The detection limit was 1 log_10_ CFU/well. The values are the means of three separate experiments. Error bars represent the standard errors. All differences were significant vs. control broth (TSB) (*p* < 0.05).

To investigate biofilm production in a more detailed manner, enumeration of bacterial burden within the biofilms was performed. For all four full-strength broths, biofilm cultures in 96-well plates were prepared. After 24 h of incubation, wells were washed with PBS, and biofilms were dispersed for CFU enumeration. For both strains, the biofilm growth estimated on the basis of bacterial cell count was the lowest in MRS broth, medium in TSB and the highest in NYC (ATCC 27956) or Schaedler broth (s. III) (Figure 1). Thus, these two broths, i.e., NYC and Schaedler broth, were chosen for further investigation focusing on the clinical strain of *S. agalactiae* (s. III).

### 2.2. CDC Biofilm Reactor System Biofilm Culture

**Figure 2:**
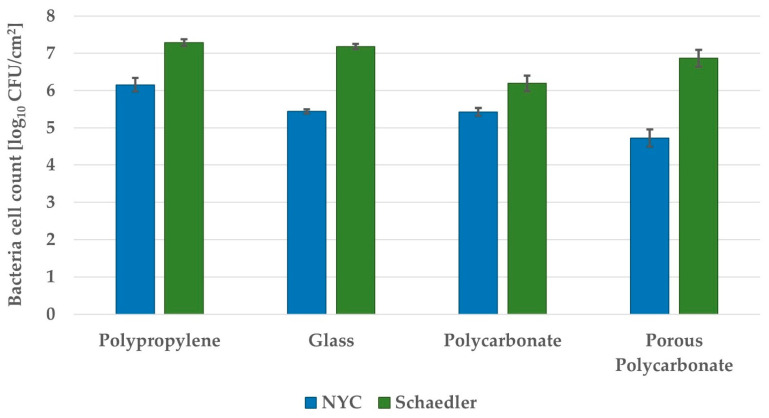
Growth of *S. agalactiae* s. III biofilm culture on coupons made from different materials in the CDC Biofilm Reactor System. The reactor with full-strength broth was inoculated with overnight culture. After 24 h of incubation with mixing, a flow of 5x-diluted broth was started and continued for 24 h. Coupons were removed and sonicated. Bacteria were plated, and colonies were enumerated. The detection limit was 2.59 log_10_ CFU/cm^2^. The values are the means of three separate experiments. Error bars represent the standard errors.

Further optimization of GBS biofilm culture required in vitro studies utilizing microtiter plates to be translated into continuous flow system culturing to mimic physiological conditions. The first step of optimization of biofilm culture under continuous flow conditions was to detect the best coupon material used as a surface for biofilm formation. Four different adherent materials were investigated, i.e., glass, polypropylene, polycarbonate, and porous polycarbonate coupons. In accordance with the results obtained for microtiter plate biofilm culture, in continuous flow biofilm culture, biofilm growth was higher in Schaedler than in NYC broth (Figure 2). In both broths, biofilm growth was the highest on polypropylene coupons. In Schaedler broth, biofilm growth was similar on polypropylene and glass coupons, lower on porous polycarbonate, and the lowest on polycarbonate coupons. In NYC broth, the highest biofilm growth was on polypropylene coupons, then lower on glass and polycarbonate coupons and the lowest on porous polycarbonate coupons.

### 2.3. Biofilm Visualization

**Figure 3:**
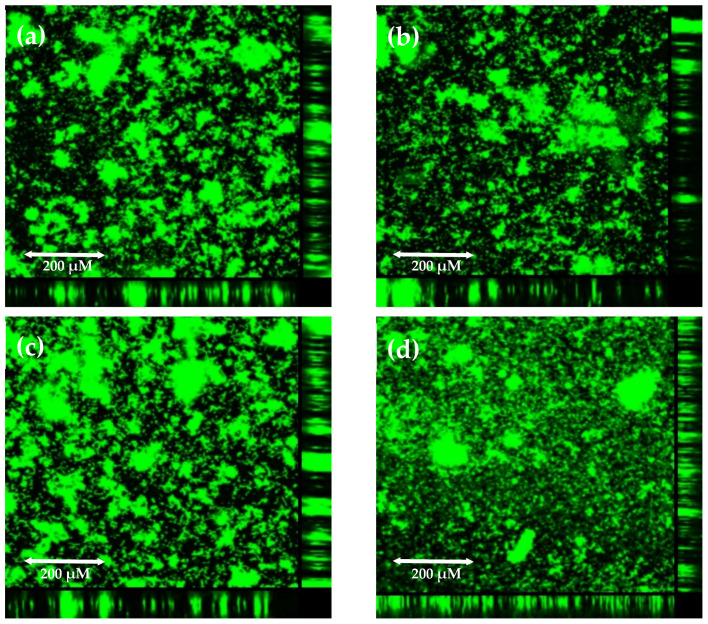
Growth of *S. agalactiae* s. III biofilm culture on coupons made from different materials in the CDC Biofilm Reactor System visualized with SYBR Green staining using confocal microscopy: (**a**) glass coupon; (**b**) polypropylene coupon; (**c**) polycarbonate coupon; (**d**) porous polycarbonate coupon. The reactor with full-strength broth was inoculated with overnight culture. After 24 h of incubation with mixing, a flow of 5×-diluted broth was started and continued for 24 h. Coupons were transferred to a glass-bottom plate, stained with SYBR Green, and viewed under a confocal laser scanning microscope.

To decide which coupons should be used for further investigation, visualization of biofilm structures using confocal microscopy was performed. Biofilm growth was visualized with SYBR Green staining. This stain intercalates into double-stranded DNA both inside and outside of the bacterial cell. For all four coupon materials, we observed mushroom-shaped microcolonies, which indicates maturity of the biofilm (Figure 3). The size and arrangement of biofilm structures are similar on all coupon materials, so this technique did not help us choose the best material; nevertheless, it was supportive to confirm mature biofilm culturing. Finally, polypropylene coupons were chosen to be the best adherent surface for use for GBS biofilm culture.

### 2.4. Evaluation of Optimized GBS Biofilm Culture with Antibacterial Treatment, i.e., aPDI

#### 2.4.1. Photodynamic Inactivation of Planktonic Cultures and Keratinocyte Safety Assays Indicate Control Conditions

**Figure 4:**
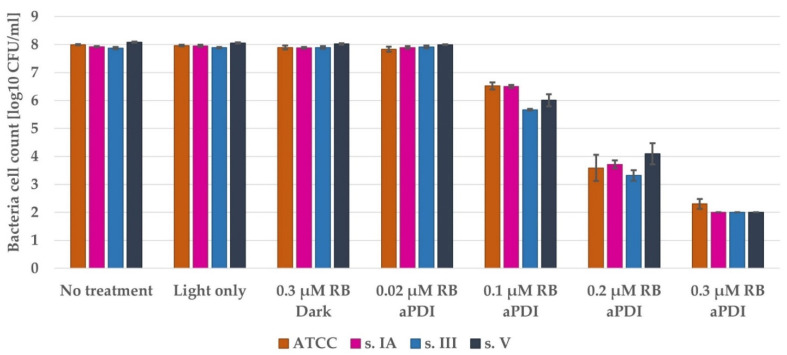
The antimicrobial effectiveness of aPDI with Rose Bengal on planktonic culture of *S. agalactiae*. An overnight culture was diluted in fresh TSB and incubated with the appropriate concentration of RB. Then, the bacteria were washed, suspended in PBS, and illuminated with a 522 nm LED lamp. After that, the bacteria were plated, and colonies were enumerated. The detection limit was 2 log_10_ CFU/mL. The values are the means of three separate experiments. Error bars represent the standard errors.

*S. agalactiae* grown in a planktonic culture is highly susceptible to RB-mediated aPDI. The concentration of 0.3 µM RB with 6 min of illumination causes ca. 6 log_10_ unit reduction in bacterial viability. Since 2 log_10_ unit CFU/mL is our limit of detection, this treatment exhibits complete eradication of all 3 clinical strains and almost complete eradication of the ATCC 27956 strain (Figure 4). Moreover, the bactericidal effectiveness was exclusively connected with the aPDI process: treatment with light only or RB in the dark did not cause a relevant reduction in bacterial viability.

**Figure 5:**
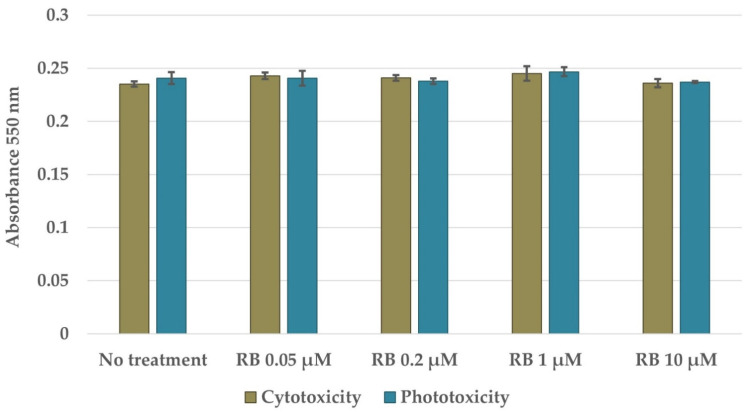
Viability of human keratinocytes (HaCaT cells) subjected to incubation with RB in the dark (cytotoxicity) or subjected to RB-mediated aPDI (phototoxicity). HaCaT cells were seeded into a 96-well plate. The next day, the cells were incubated in dark with RB or incubated in dark with RB and then illuminated. The following day, the cells were incubated with MTT compound and lysed with DMSO for absorbance measurement. The values are the means of three separate experiments. Error bars represent the standard deviations.

To indicate whether the treatment conditions could exert bactericidal effects with limited harmful activity toward human keratinocytes, the cytotoxicity and phototoxicity of the treatment conditions were assayed using the MTT test. Rose Bengal manifested no cytotoxic activity against HaCaT cells at concentrations up to 10 µM (Figure 5). Moreover, aPDI under the evaluated illumination conditions was safe for HaCaT cells across the whole analyzed RB concentration spectrum.

#### 2.4.2. Photodynamic Inactivation of Microtiter Plate Biofilm Cultures

**Figure 6:**
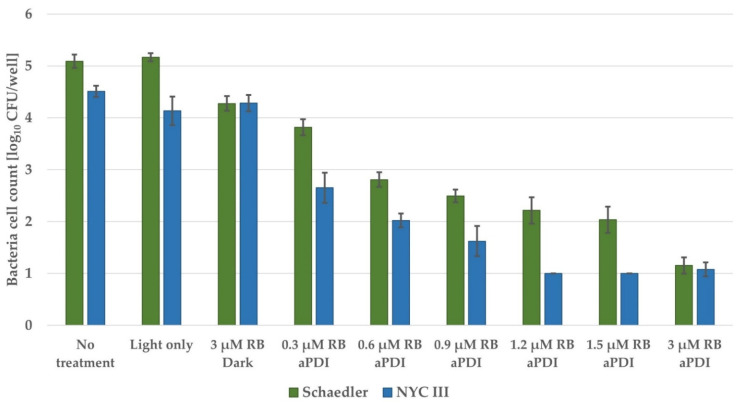
The antimicrobial effectiveness of aPDI with Rose Bengal on biofilm culture of *S. agalactiae* s. III grown on microtiter plates. An overnight culture was diluted in appropriate medium and incubated for 4 h. Then, the broth was changed, and incubation continued for 20 h. The biofilm was incubated with an appropriate concentration of RB. Then, the biofilm was washed, suspended in PBS, and illuminated with a 522 nm LED lamp. Dispersed biofilms were plated, and colonies were enumerated. The detection limit was 1 log_10_ CFU/well. The values are the means of three separate experiments. Error bars represent the standard errors.

It is commonly known that microorganisms living in biofilms are more tolerant to various antibacterial treatments. The same could be observed for aPDI treatment. Therefore, to detect the most effective aPDI conditions, we screened multiple photosensitizer concentrations starting with the highest concentration used for planktonic studies. For NYC broth, a reduction in bacterial viability reaching the limit of detection was observed when RB was administered at a concentration of 1.2 µM (Figure 6). In the case of Schaedler broth, a similar effect was reached by employing RB at a concentration 10 times higher than that for planktonic culture (3 µM). Since the total initial number of bacteria in biofilms was much lower than that for planktonic culture, the viability decrease was 4.5 and 5 log_10_ units CFU/well for NYC and Schaedler broths, respectively. Similar to planktonic culture, light-only treatment did not cause any decrease in bacterial viability, and RB in the dark treatment exhibited limited toxicity in the dark (only in the case of biofilms formed in Schaedler broth), leading to a reduction in bacterial viability by ca. 1 log_10_ unit CFU/well. The obtained data also indicate that the biofilm grown in Schaedler broth is much more tolerant to aPDI than that grown in NYC medium.

#### 2.4.3. Photodynamic Inactivation of CDC Biofilm Reactor System Biofilm Culture

**Figure 7:**
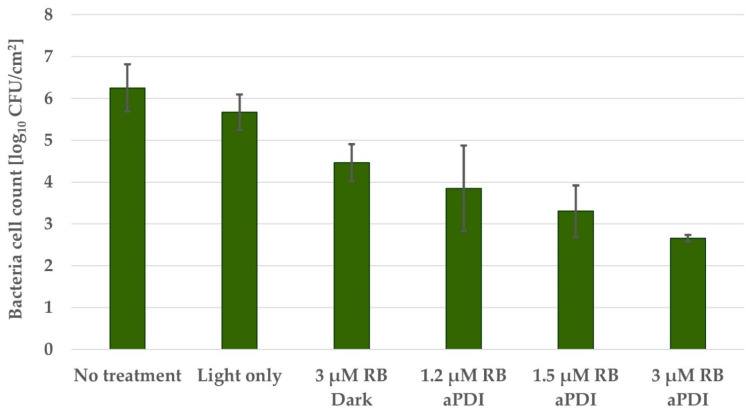
The antimicrobial effectiveness of aPDI with Rose Bengal on biofilm culture of *S. agalactiae* s. III grown in Schaedler medium on polypropylene coupons in the CDC Biofilm Reactor System. The reactor with full-strength broth was inoculated with overnight culture. After 24 h of incubation with mixing, a flow of 5× diluted broth was started and continued for 24 h. Coupons were removed and incubated with an appropriate concentration of RB. Then, the biofilm was washed, suspended in PBS, and illuminated with a 522 nm LED lamp. Coupons were then sonicated. Bacteria were plated, and colonies were enumerated. The detection limit was 2.59 log_10_ CFU/cm^2^. The values are the means of three separate experiments. Error bars represent the standard errors.

For biofilms grown in a continuous flow system, the three most effective RB concentrations were used. Since the coupon surface is 2.53 cm^2^, the limit of detection was 2.59 log_10_ CFU/cm^2^. aPDI with 3 µM RB caused a bacterial viability reduction of 3.6 log_10_ unit CFU/cm^2^ (Figure 7), reaching the limit of detection. Similar to biofilm culture on microtiter plates, light-only treatment did not cause a decrease in bacterial viability, and RB treatment in the dark caused a toxicity effect reaching a viability reduction of 1.8 log_10_ unit CFU/cm^2^. This is the first time that toxicity of RB in the dark has been observed, and its explanation requires further investigation.

## 3. Discussion

There is no single recommended method for *S. agalactiae* biofilm growth experiments. *S. agalactiae* biofilms are usually grown on polystyrene 96-well plates in TSB [21,22,23,24] or THB [6,25,26,27,28,29,30]. Some deviations have been reported, such as THB supplemented with yeast extract [31], TSB supplemented with bovine serum [5], TSB supplemented with 3% BSA [32], RPMI [27], cation adjusted—Mueller Hinton broth (CA-MHB) [33] or CA-MHB supplemented with lysed horse broth [34]. Erika C. R. Bonsaglia et al. compared biofilm growth in four different broths, i.e., TSB, THB, LB, and BHI, among which growth in TSB was the greatest [8]. There has also been an attempt to culture *S. agalactiae* biofilms in simulated vaginal fluid, but biofilm growth was still better in TSB [7]. Therefore, our observation that *S. agalactiae* biofilm formation in NYC or Schaedler broth is greater than that in TSB should be beneficial for further model unification.

As the vaginal environment is affected by the frequent flow of fluids, we concluded that a biofilm model grown in continuous flow conditions, as in the CDC reactor, would better imitate the natural situation. Additionally, Buckingham–Meyer, Goeres, and Hamilton demonstrated that *Pseudomonas aeruginosa* and *Staphylococcus aureus* biofilms grown in a CDC reactor were more resistant to commonly used disinfectants than biofilms grown in static conditions [35]. They recommended this method as a model for the measurement of disinfectant efficacy. To date, *S. agalactiae* biofilms have been cultured only under static conditions on polystyrene plates or in a Calgary Biofilm Device [5], in which pegs are submerged in 96-well plates containing broth and can be exposed to shaking. In this work, we undertook the first attempt to culture *S. agalactiae* biofilms under continuous flow conditions. Previously, biofilms of *Streptococcus pneumoniae* [36] and *Streptococcus mutans* [37] have been grown in a CDC reactor. *S. pneumoniae* biofilms have been grown on polycarbonate coupons in BHI broth supplemented with casein and yeast extract. The batch phase was set for 12 h, while the flow phase with 10% BHI broth with supplements was set for 24 h. For *S. mutans* biofilm culture, hydroxyapatite coupons were used, and 1% TSB was used in both phases. Both phases were also set for 24 h. In our protocol for *S. agalactiae* biofilm culture, we concluded that the use of polypropylene coupons and Schaedler broth leads to the growth of the highest number of bacteria. Both phases were set for 24 h, and in the flow phase, we used 20% Schaedler broth. Apart from the high number of bacterial cells, we also observed that in our model biofilms grew as mushroom-shaped microcolonies, which we previously described as indicative of biofilm maturity [38]. It was important for us to evaluate biofilm growth on different coupon materials because previous studies have shown that some surfaces allow for a better biofilm growth support i.e., Teflon for *Candida albicans* [39], zinc-galvanized steel for *Mycobacterium* sp. [40], or stainless steel for *Flavobacterium psychrophilum* [41]. Moreover, Dustin L. Williams et al. showed that change of coupon material from polycarbonate to collagen influences biofilm susceptibility to antibiotics, which can be an interesting direction for future biofilm model improvements [42]. Schaedler broth has a much richer composition than TSB. Schaedler has all the ingredients of TSB, but with a higher concentration of glucose (0.5% vs. 0.25%) which promotes biofilm growth of, i.e., *Staphylococcus aureus* and *Straphylococcus epidermidis* [43]; addition of animal tissue peptone which increases the content of available amino acids; addition of yeast extract which is necessary for biofilm growth of some bacteria, i.e., *Actinobacillus succinogenes* [44], but is also an additional source of vitamins; addition of cysteine, which is important for biofilm growth of i.e., *Streptococcus mutans* [45] and addition of haemin which is an iron source favoring more complex biofilm growth of i.e., *Actinobacillus actinomycetemcomitans* [46]. NYC is not as rich as Schaedler but has a richer composition than TSB. NYC, in comparison to TSB, has animal tissue peptone instead of tryptone (derived from casein) and soytone (derived from soya) but has additional yeast extract and the same glucose concentration as Schaedler broth. MRS also has a richer composition than TSB. A much higher concentration of glucose in MRS in comparison to TSB (2% vs 0.25%) may be the reason why biofilm growth is lower in MRS than in TSB. It was previously observed that high glucose concentration inhibits biofilm formation of, i.e., *Aeromonas hydrophila* [47].

Because of the high antibiotic resistance of biofilm-embedded cells, there is a tremendous demand for the evaluation of alternative approaches [5,31]. Against *S. agalactiae* biofilms, therapies based on human milk oligosaccharides [25,26], tea saponin [21], benzalkonium chloride [24], staphylococcal bacteriophage lysin CHAPk [32], or synthetic ellagic acid glycosides [29] have been proposed. Therapies based on the synergistic effect of antibiotics [34], synergistic effect of silver nanoparticles with eugenol [23], or silver nanoparticles with cinnamon oil [22] have also been proposed. We have previously summarized the use of photoinactivation against biofilms of ESKAPE pathogens [48], which cause numerous clinical infections. There have been few attempts of photoinactivation use against *S. agalactiae* planktonic culture [49,50,51,52,53]; however, there have been no reports of the use of photoinactivation against *S. agalactiae* biofilms. We are happy to report that Rose Bengal-mediated photoinactivation can be successfully used against *S. agalactiae* biofilms in both static and continuous flow models. As we expected, eradication of biofilm cultures requires the use of much higher concentrations of RB than planktonic culture. Additionally, biofilms grown in a continuous flow system require the use of higher RB concentrations than biofilms grown in static conditions, which confirms the success of biofilm culture optimization and new model introduction. The necessity of more rigorous photoinactivation conditions application for eradication of biofilm grown in Schaedler broth in comparison to NYC broth may result from the presence of L-cystine in Schaedler broth. L-cysteine is a reducing agent and plays role in the detoxification of hydrogen peroxide [54]. Nevertheless, in our opinion presence of L-cysteine mimics a better vaginal environment, because it is present in healthy women’s vaginas and is crucial for the growth of physiological flora, i.e., *Lactobacillus iners* [55]. Since RB-mediated aPDI is safe for human keratinocytes, it is a promising therapeutic approach against *S. agalactiae* biofilms, which should be investigated in-depth in the future.

## 4. Materials and Methods

### 4.1. Bacterial Strains and Culture Media

In this study, 4 strains of *Streptococcus agalactiae* were used (ATCC 27956 and 3 clinical strains: 1030/06, 2306/06, and 2974/07, representing serotypes IA, III, and V, respectively). The clinical strains were kindly provided by Izabela Sitkiewicz, National Medicines Institute, Warsaw, Poland). Columbia blood agar plates (Biomerieux, Craponne, France) were used for colony-forming unit (CFU) determination. Tryptic soy broth (TSB) (Biomerieux, Craponne, France) was used for overnight planktonic culture, and TSB, De Man, Rogosa and Sharpe (MRS) broth (BTL, Lodz, Poland), New York City III broth (NYC), and Schaedler (Oxoid, Basingstoke, UK) broth were used for biofilm culture. NYC broth was prepared on-site, containing HEPES (Sigma Aldrich, Saint Louis, MO, USA), proteose peptone (Sigma Aldrich, USA), yeast extract (Pol-Aura, Roznowo, Poland), sodium chloride (Stanlab, Lublin, Poland), and anhydrous glucose (Chempur, Piekary Slaskie, Poland).

### 4.2. Photosensitizing Agents

4,5,6,7-Tetrachloro-2′,4′,5′,7′-tetraiodofluorescein disodium salt (Rose Bengal, RB) powder was purchased from Sigma (Sigma-Aldrich, Saint Louis, MO, USA). A stock solution (10 mM) was prepared in Millipore distilled water and kept in the dark at 4 °C.

### 4.3. Light Source

A custom constructed LED-based light source was used, which emitted λmax 522 nm light with a radiosity of 10.6 mW/cm^2^ (FWDH (full width half maximum): 34 nm) (Cezos, Gdynia, Poland).

### 4.4. Microtiter Plate Biofilm Culture

Biofilms were cultured on 96-well flat-bottom microtiter plates (Nest Biotechnology, Wuxi, Jiangsu, China). Four different broths (TSB, MRS, NYC, and Schaedler) were used in complete formula or diluted two times in double-distilled water. An overnight *S. agalactiae* culture was diluted 20 times in the appropriate broth (initial bacterial inoculum 10^7^ CFU/mL), and 200 µL aliquots were transferred into plates in three technical repetitions. The negative control was broth without bacteria. The plate was covered with sealing tape and incubated at 37 °C for 4 h. Then, the medium was removed, replaced with 200 µL of fresh appropriate broth, and incubated at 37 °C for 20 h. The experiment was conducted in three replicates.

### 4.5. Crystal Violet Staining

Biofilm cultures were washed three times with phosphate-buffered saline (PBS) (Sigma-Aldrich, Saint Louis, MO, USA) and fixed with 2% sodium acetate for 15 min. Then, the cells were stained with 0.1% crystal violet for 20 min and washed three times with distilled water. After drying, the precipitate was resolved in freshly prepared 33% acetic acid, and the absorbance at 570 nm was measured using an EnVision plate reader (Perkin Elmer, Waltham, MA, USA). The optical density of the samples (OD), which corresponds to the adhesion ability of the biofilm, was compared with the optical density of the negative control (ODc). The ODc was calculated as the mean of the negative control absorbance with the addition of three times the SD value. The following classification was used for the determination of biofilm production: nonadherent (OD ≤ ODc), weakly adherent (ODc < OD ≤ 2 ODc), moderately adherent (2 ODc < OD ≤ 4 ODc), and strongly adherent (4 ODc < OD).

### 4.6. CDC Biofilm Reactor System Biofilm Culture

For biofilm culture, a CDC biofilm reactor model (BioSurface Technologies, Bozeman, MT, USA) was used. Coupons made of glass, polypropylene, polycarbonate, and porous polycarbonate were used. Before each culture, a whole setup was prepared as previously described [56]. Sterile broth (NYC or Schaedler) in the reactor was inoculated with 1 mL of 2.4 McFarland units (Densi-La-Meter II, ERBA Lachema, Brno, Czech Republic) adjusted overnight culture of *S. agalactiae.* The reactor was placed onto a magnetic stirrer with a heater set at 80 rpm and 37 °C for 24 h, which was the batch phase. Before starting the flow phase, 1 L of 4× concentrated sterile broth (NYC or Schaedler) was added to a 20 L carboy containing 19 L of distilled water autoclaved for 2 h at 14.7 psi. The final concentration of broth was five times lower than that recommended by the manufacturer. The carboy was connected to the reactor by silicone tubing and connected to a peristaltic pump (Watson–Marlow Fluid Technology Group, Falmouth, UK). The flow rate was set to 10.8 mL/min, and the reactor volume was 335 mL, which resulted in a residence time of 31 min, which was shorter than the *S. agalactiae* generation time. The time of the flow phase was set for 24 h. For CFU determination, the coupons were transferred to 15 mL Falcon tubes containing 10 mL of PBS. The coupons were sonicated for 1 min (Ulsonix, Proclean 3.0 DSP, Expando, Berlin, Germany), vortexed for 1 min and incubated on ice for 1 min. The whole procedure was repeated three times. Then, bacteria dispersed from the coupons were serially diluted in PBS and transferred onto Columbia blood agar plates. After 18 h of incubation at 37 °C, colonies were enumerated, and CFU/mL values were determined. The experiment was conducted in three replicates.

### 4.7. Biofilm Visualization

Biofilm growth on coupons was visualized using confocal microscopy. Visualization of biofilms was performed with SYBR Green staining. Coupons were washed in PBS and transferred to a 12-well glass-bottom plate containing 500 µL of PBS and incubated in the presence of 2 µL of 100× concentrated SYBR Green for 15 min in the dark at RT. Specimens were imaged using a confocal laser scanning microscope (Leica SP8X) with a 10× lens (Leica Biosystems, Nussloch, Germany). During observation, the excitation wavelength was 488 nm, and the emission wavelength range used for detecting SYBR Green was 501–548 nm. Photographs were obtained and then analyzed with Leica LAS X software.

### 4.8. Photodynamic Inactivation of Planktonic Cultures

An overnight culture (1 colony transferred into 5 mL of TSB and incubated for 18 h at 37 °C with shaking at 150 rpm) of *S. agalactiae* was adjusted to 2.4 McFarland units in PBS, which corresponds to a cell density of approx. 10^8^ CFU/mL. Working solutions of RB were prepared in Millipore distilled water. A total of 180 µL of bacterial suspension and 20 µL of photosensitizer solution were mixed in Eppendorf tubes and incubated in the dark at 37 °C for 15 min. Then, the samples were washed twice, centrifuged (10,000× *g*, 3 min), and resuspended in PBS. Aliquots of 100 µL of each sample were transferred into a 96-well plate (Nest Biotechnology, Wuxi, Jiangsu, China) and illuminated with a 522 nm LED lamp for 6 min (3.8 J/cm^2^). Afterward, samples were serially diluted in PBS and transferred onto Columbia blood agar plates. After 18 h of incubation at 37 °C, the colonies were counted, and CFU/mL values were determined. The experiment was conducted in three replicates.

### 4.9. Photo- and Cytotoxicity Assays Based on MTT

Photo- and cytotoxicity assays were previously described [57]. Briefly, HaCaT cells (CLS 300493) were seeded the day before treatment in three biological replicates for each condition in two 96-well plates (for light and dark conditions). The cells were grown in a humidified incubator at 37 °C and in a 5% CO_2_ atmosphere in supplemented high-glucose DMEM (Life Technologies/Thermo Fisher Scientific, Waltham, MA, USA). RB was added directly to the medium and incubated for 15 min at 37 °C. Then, the cells were washed twice with PBS, and 100 μL of fresh medium was added. Next, the cells were illuminated with a 522 nm LED lamp for 6 min (3.8 J/cm^2^). Cell survival was measured after 24 h of incubation at 37 °C by an MTT [3-(4,5-dimethylthiazol-2-yl)-2,5-diphenyltetrazolium bromide] assay. Briefly, 10 μL of an MTT solution (12 mM) was applied to each well and incubated for 4 h at 37 °C. The cells were then lysed in DMSO (Sigma-Aldrich, Saint Louis, MO, USA), and the absorbance of the formazan was measured at 550 nm using an EnVision plate reader (Perkin Elmer, Waltham, MA, USA).

### 4.10. Photodynamic Inactivation of Microtiter Plate Biofilm Cultures

Biofilms cultured in NYC or Schaedler broth were washed with PBS and then incubated with different RB concentrations in PBS in the dark at 37 °C for 15 min. Then, the biofilms were washed twice with PBS and illuminated for 6 min. After illumination, the biofilms were dispersed by scraping with a pipette tip and thorough pipetting. Afterward, samples were serially diluted in PBS and transferred onto Columbia blood agar plates. After 18 h of incubation at 37 °C, the colonies were counted, and CFU/mL values were determined. The experiment was conducted in three replicates.

### 4.11. Photodynamic Inactivation of CDC Biofilm Reactor System Biofilm Culture

Coupons were washed in PBS and transferred to a 12-well plate containing 1.5 mL of PBS with/without RB and incubated in the dark for 15 min at 37 °C. Then, the coupons were transferred to another 12-well plate containing 1.5 mL of PBS and illuminated on both sides for 6 min on each side. Then, the coupons were transferred to 15 mL Falcon tubes containing 10 mL of PBS. The coupons were sonicated for 1 min, vortexed for 1 min, and incubated on ice for 1 min. The whole procedure was repeated three times. Then, bacteria dispersed from the coupons were serially diluted in PBS and transferred onto Columbia blood agar plates. After 18 h of incubation at 37 °C, colonies were enumerated, and CFU/mL values were determined. The experiment was conducted in three replicates.

### 4.12. Statistical Analysis

The statistical analyses were performed using Excel. The quantitative variables were characterized by the arithmetic mean of standard deviation. Statistical significance of differences between two groups was processed with the Student’s *t*-test. In all calculations, a statistical significance level of *p* < 0.05 was used.

## 5. Conclusions

*Streptococcus agalactiae* biofilm formation depends on the used medium and surface material. We believe that a continuous flow biofilm model is a better representation of the vaginal environment than a static biofilm model and we recommend the application of this model in future antibacterial studies. This model was successfully applied in photoinactivation studies and allowed to show its effectiveness. RB-mediated photoinactivation is effective in the eradication of both planktonic and biofilm cultures of *S. agalactiae*. It is also safe for human keratinocytes which makes it a promising antimicrobial therapy.

## Figures and Tables

**Table 1:** Results of *S. agalactiae* biofilm production assessed with crystal violet staining.

	Medium Type							
*S. agalactiae* strain	TSB		MRS		NYC		Schaedler	
	0.5×	1×	0.5×	1×	0.5×	1×	0.5×	1×
ATCC 27956	++	+	++	+	++	++	++	++
s. IA	++	+	++	+	++	+	+	+
s. III	+/−	+/−	++	+	++	++	++	++
s. V	+/−	+/−	+/−	+/−	−	+/−	+/−	+/−

Legend: (−) nonadherent; (+/−) weakly adherent; (+) moderately adherent; (++) strongly adherent.

## Data Availability

The datasets generated and/or analyzed during the current study are available from the corresponding author on reasonable request.
